# The association between obesity, health service use, and work productivity in Australia: a cross-sectional quantile regression analysis

**DOI:** 10.1038/s41598-023-33389-4

**Published:** 2023-04-24

**Authors:** Marie Ishida, Monique D’Souza, Yang Zhao, Tianxin Pan, Will Carman, Tilahun Haregu, John Tayu Lee

**Affiliations:** 1 School of Population and Global Health, Nossal Institute for Global Health, University of Melbourne, Melbourne, Australia; 2 The George Institute for Global Health at Peking University Health Science Center, Beijing, China; 3 The George Institute for Global Health at University of New South Wales, Sydney, Australia; 4 Health Economics Unit, Centre for Health Policy, Melbourne School of Population and Global Health, University of Melbourne, Melbourne, Australia; 5 Department of Primary Care and Public Health, Faculty of Medicine, Imperial College London, London, UK; 6 Department of Health Service Research, Faculty of Medicine, Australian National University, Canberra, Australia

**Keywords:** Health services, Public health, Health policy

## Abstract

The burden of disease attributable to obesity is rapidly increasing and becoming a public health challenge globally. Using a nationally representative sample in Australia, this study aims to examine the association of obesity with healthcare service use and work productivity across outcome distributions. We used Household, Income and Labour Dynamics Australia (HILDA) Wave 17 (2017–2018), including 11,211 participants aged between 20 and 65 years. Two-part models using multivariable logistic regressions and quantile regressions were employed to understand variations in the association between obesity levels and the outcomes. The prevalence of overweight and obesity was 35.0% and 27.6%, respectively. After adjusting for socio-demographic factors, low socioeconomic status was associated with a higher probability of overweight and obesity (Obese III: OR = 3.79; 95% CI 2.53–5.68) while high education group was associated with a lower likelihood of being high level of obesity (Obese III OR = 0.42, 95% CI 0.29–0.59). Higher levels of obesity were associated with higher probability of health service use (GP visit Obese, III: OR = 1.42 95% CI 1.04–1.93,) and work productivity loss (number of paid sick leave days, Obese III: OR = 2.40 95% CI 1.94–2.96), compared with normal weight. The impacts of obesity on health service use and work productivity were larger for those with higher percentiles compared to lower percentiles. Overweight and obesity are associated with greater healthcare utilisation, and loss in work productivity in Australia. Australia’s healthcare system should prioritise interventions to prevent overweight and obesity to reduce the cost on individuals and improve labour market outcomes.

## Introduction

The growing prevalence of obesity and overweight has become a global public health concern. Globally, the prevalence of obesity has doubled since 1980^1^. The Global Burden of Disease group (2017) estimated that high body mass index (BMI) contributed to 4.0 million deaths, which represented 7.1% of the deaths from any cause; it also contributed to 120 million disability-adjusted life-years (DALYs), which represented 4.9% of DALYs from any cause among adults globally^1^. Excessive body weight (including overweight and obesity) was associated with 8.4% of the overall health burden in Australia (2020)^2^. It is currently estimated that 2 in 3 Australian adults are overweight or obese^2^. Furthermore, there has been a worrying shift in the proportion of those who are ‘overweight’ to ‘obese’^3^. For example, while the prevalence of overweight has somewhat stabilised over the past three decades, there has been a large increase in prevalence of obesity from 18.7% in 1995 to 31.3% in 2018^4^. The prevalence of overweight and obesity also follows a socioeconomic gradient, being more common in lower socio-economic groups and among the Indigenous population^2^. National statistics found that female Indigenous Australians are 1.2 times more likely to be overweight and 1.5 times more likely to be obese in comparison to their non-Indigenous counterparts^2^.

The rising burden of overweight and obesity has an impact on health systems and national economies. There are few studies investigating the economic impact of excessive weight on health service use, direct medical cost and productivity loss but most of them are from US or Europe^5–8^. These studies have demonstrated that higher level of BMI is associated with increased number of outpatient visits, emergency room visits, higher probability of hospitalisation, higher level of health expenditures and work productivity loss^9–12^.

The literature on the economic impact of overweight and obesity is extremely small compared to the rapid increase in prevalence of obesity in Australia. Existing studies examined the association between high BMI and health costs^13–15^. For example, Colagiuri and colleagues (2010) analysed 5-year follow-up data from the Australian Diabetes, Obesity and Lifestyle study and found that the annual total direct cost per person increased from $1472 for those of normal weight to $2788 for the obese in 2005^13^. Brown and colleagues (2008) examined relationships between physical activity and BMI with health care costs in women. They reported that higher BMI was associated with increased risk of making more than 15 claims per year with the national Medicare health insurance^16^. There are two studies that examined the impact of high BMI on productivity loss in Australia, including one focused on Australian defence force personnel^17^, and one estimated the societal benefit of reducing obese together with five other risk factors in Australia^18^. However, these studies are limited in selected geographical areas or a subgroup of the populations. Little is known on the impact of overweight and obesity on health service use and productivity loss among general population in Australia. In addition, previous studies in Australia have utilised traditional linear regression, with a focus on the mean effect of obesity on economic outcomes. However, the association between overweight and obese and economic outcomes (such as service use, health cost and productivity loss) may vary across the distribution of outcomes. Quantile regression provides a more robust estimation on the effect between chronic conditions, risk factors and economic outcome across population^19^. To fill this important evidence gap, using a nationally presentative data, we present the first study in Australia that applied quantile regression methods to examine the effect of overweight and obesity on health service utilisation and labour force participation.

## Methods

### Sample and data

We used the nationally representative cross-sectional data from the Household, Income and Labour Dynamics in Australia (HILDA) wave 17, which was conducted between 2017 and 2018. HILDA is a nationally representative longitudinal study of Australian residents aged 15 years and above. The baseline survey of HILDA commenced in 2001, collecting information annually on various aspects of life in Australia, including demographics, socio-economic status, healthcare utilisation, work productivity, and the Health-Related Quality of Life (HRQoL). Multi-stage sampling approach was used to select sample households. First, a stratified random sample of 488 Census Collection District containing 200 to 250 households in each district was selected^20^. A sample of 22 to 34 dwellings were then randomly selected, and within each dwelling, up to three random households were selected^20^. Interviews and self-completed questionnaire following the University of Melbourne’s ethical guidelines were used for data collection. Further detailed description of the survey objectives and data collection method can be found elsewhere^20^. HILDA Wave 17 includes a total of sample including 17,570 respondents with a response rate of 96.4%. In this analysis, we included working-age respondents between 20 and 65 years old with Body Mass Index (BMI) ≥ 18.5 kg/m^2^ and excluded those who had a missing value on outcomes and any of the covariates. The final sample consists of 11,211 respondents.

### Measures obesity levels

The survey provided respondents’ BMI, which was derived from self-reported weight in kilograms and height in meters (kg/m^2^). We classified BMI into five obesity levels according to the World Health Organisation (WHO) definition; normal weight (BMI: 18.5–24.9 kg/m^2^), overweight (BMI: 25–29.9 kg/m^2^), obesity class I (BMI: 30–34.9 kg/m^2^), obesity class II (BMI: 35–39.9 kg/m^2^), and obesity class III (BMI: ≥ 40 kg/m^2^)^21^.

### Outcomes

In this study, health service use was measured using the number of General Practitioner (GP) visits and nights spent at hospital, as well as the number of prescribed medications for regular use in the previous 12 months. Work productivity was measured through the number of paid sick leave days taken in the last 12 months for the respondents who were employed at the time of the survey.

### Covariates

Covariates included sex, age group (20–29, 30–39, 40–49, 50–59, 60–65), education level (low, middle, high), household income (1–4 quantiles of sample), Indigenous status (Non-Indigenous Australian, Indigenous Australian), country of birth (Australia, main English-speaking countries, other), marital status (married/de facto, others), Australian state (New South Wales, Victoria, South Australia, Queensland, Western Australia, Tasmania, Northern Territory, Australian Capital Territory), residential area (urban, rural), alcohol intake (no alcohol intake, drink alcohol less than 3 days per week, drink alcohol 3 and more days per week), smoking (non-smoker, ex-smoker, current smoker).

### Statistical analysis

We examined the determinants of overweight and obesity using multivariable logistic regression models. Two-part models were used to investigate the association of overweight and obesity with health service use and work productivity loss. First, we applied a multivariable logistic regression model to examine the probability of observing positive binary outcomes for any health service use (yes/no) and any sick leave taken (yes/no) against obesity levels. Second, for those with positive binary outcomes for these variables, we performed multivariable quantile regression to estimate the different impacts of overweight and obesity on health service use and loss of work productivity across their distributions (10th, 25th, 50th, 75th, and 90th percentiles). The quantile regression is estimating the quantiles for the outcome variable associated with a set of independent variables and covariates without assuming normality or homoscedasticity of the underlying distribution^22,23^. Advantage of the quantile regression is the robustness to outliers as it allows for assessing the full distribution of the outcome variable and is suitable for modelling outcomes that are not normally distributed or are highly skewed^22,23^.

In this study, the coefficients at the lower percentiles of the health service use distribution (e.g. the 10th, 20th, and 30th percentiles) indicate the association between obesity and low level of health service use, while the coefficient at the higher percentiles of the health service use distributions (e.g. 70th, 80th, and 90th percentiles) reflect the association between overweight and obesity and high level of health service use. In addition, we performed Ordinary Least Square models (OLS) and presented estimated mean change of the outcomes by overweight and obesity levels to compare with the estimated change at different points of outcome distributions obtained from multivariable quantile regression models. All multivariable regression models were adjusted for covariates listed above and *p*-values < 0.05 were considered statistically significant. All analyses were performed using Stata 15 (Stata Corp., College Station, Texas).

## Results

### Sample characteristics

The study sample consisted of 11,211 respondents. The median age of respondents was 42 (IQR 30–53) and BMI was 26.4 (IQR 23.4–30.5). The majority of respondents were married/de facto (70.6%), living in an urban area (87.4%), with a middle or high education level (84.1%). The prevalence of each obesity level was 37.5% (normal weight), 35.0% (overweight), 16.8% (obese I), 6.69% (obese II), and 4.08% (obese III). (Table 1).

**Table 1:** Sample characteristics.

	Total		Normal weight	Overweight	Obese I	Obese II	Obese III
	N	%	N (%)	N (%)	N (%)	N (%)	N (%)
Overall	11,211	100	4206 (37.5)	3920 (35.0)	1878 (16.8)	750 (6.69)	457 (4.08)
Sex							
Male	5342	47.7	1693 (40.3)	2254 (57.5)	947 (50.4)	300 (40.0)	148 (32.4)
Female	5869	52.4	2513 (59.8)	1666 (42.5)	931 (49.6)	450 (60.0)	309 (67.6)
Income							
Lowest	2804	25.0	1071 (25.5)	858 (21.9)	489 (26.0)	222 (29.6)	164 (35.9)
2nd quantile	2802	25.0	995 (23.7)	977 (24.9)	515 (27.4)	188 (25.1)	127 (27.8)
3rd quantile	2803	25.0	1044 (24.8)	1003 (25.6)	469 (25.0)	186 (24.8)	101 (22.1)
Highest	2802	25.0	1096 (26.1)	1082 (27.6)	405 (21.6)	154 (20.5)	65 (14.2)
Age							
20–29	2629	23.5	1314 (31.2)	762 (19.4)	342 (18.2)	131 (17.5)	80 (17.5)
30–39	2484	22.2	989 (23.5)	888 (22.7)	373 (19.9)	141 (18.8)	93 (20.4)
40–49	2378	21.2	777 (18.5)	880 (22.5)	422 (22.5)	174 (23.2)	125 (27.4)
50–59	2431	21.7	724 (17.2)	920 (23.5)	484 (25.8)	192 (25.6)	111 (24.3)
60–65	1289	11.5	402 (9.56)	470 (12.0)	257 (13.7)	112 (14.9)	48 (10.5)
Education							
Low education	1781	15.9	531 (12.6)	570 (14.5)	391 (20.8)	178 (23.7)	111 (24.3)
Middle education	5821	51.9	2034 (48.4)	2045 (52.2)	1944 (55.6)	425 (56.7)	273 (59.7)
High education	3609	32.2	1641 (39.0)	1305 (33.3)	443 (23.6)	147 (19.6)	73 (16.0)
Indigenous status							
Non-Indigenous	10,912	97.3	4126 (98.1)	3824 (97.6)	1808 (96.3)	714 (95.2)	440 (96.3)
Indigenous	299	2.67	80 (1.9)	96 (2.45)	70 (3.73)	36 (4.80)	17 (3.72)
Country of birth							
Australia	9019	80.5	3228 (77.0)	3161 (80.6)	1584 (84.4)	638 (85.1)	398 (87.1)
Main English speaking country	936	8.35	330 (7.85)	344 (8.78)	155 (8.25)	72 (9.6)	35 (7.66)
Others	1256	11.2	638 (15.2)	415 (10.6)	139 (7.40)	40 (5.33)	24 (5.25)
Marital status							
Married/de facto	7917	70.6	2844 (67.6)	2895 (73.9)	1370 (73.0)	517 (68.9)	291 (63.7)
Others	3294	29.4	1362 (32.4)	1025 (26.2)	508 (27.1)	233 (31.1)	166 (36.3)
State							
New South Wales	3184	28.4	1239 (29.5)	1135 (29.0)	507 (27.0)	199 (26.5)	104 (22.8)
Victoria	2839	25.3	1120 (26.6)	986 (25.2)	447 (23.8)	171 (22.8)	115 (25.2)
Queensland	2479	22.1	898 (21.4)	864 (22.0)	435 (23.2)	172 (22.9)	110 (24.1)
South Australia	999	8.91	349 (8.3)	311 (7.93)	195 (10.4)	83 (11.1)	61 (13.4)
Western Australia	980	8.74	345 (8.2)	372 (9.49)	154 (8.20)	71 (9.47)	38 (8.32)
Tasmania	379	3.38	116 (2.76)	130 (3.32)	79 (4.21)	34 (4.53)	20 (4.4)
Northern Territory	99	0.88	39 (0.93)	37 (0.94)	14 (0.75)	6 (0.80)	3 (0.7)
Australian Capital territory	252	2.25	100 (2.38)	85 (2.17)	47 (2.50)	14 (1.87)	6 (1.3)
Area							
Urban	9801	87.4	3755 (89.3)	3408 (86.9)	1610 (85.7)	633 (84.4)	395 (86.4)
Rural	1410	12.6	451 (10.7)	512 (13.1)	268 (14.3)	117 (15.6)	62 (13.6)
Alcohol intake							
No intake	4388	39.1	1594 (37.9)	1332 (34.0)	817 (43.5)	373 (49.7)	272 (59.5)
Drink alcohol < 3 days per week	4016	35.8	1577 (37.5)	1450 (37.0)	611 (32.5)	248 (33.1)	130 (28.5)
Drink alcohol 3 + days per week	2807	25	1035 (24.6)	1138 (29.0)	450 (24.0)	129 (17.2)	55 (12.0)
Smoking							
Non-smoker	6110	54.5	2573 (61.2)	2111 (53.9)	865 (46.1)	342 (45.6)	219 (47.9)
Ex smoker	2940	26.2	857 (20.4)	1097 (28.0)	602 (32.1)	246 (32.8)	138 (30.2)
Current smoker	2161	19.3	776 (18.5)	712 (18.2)	411 (21.9)	162 (21.6)	100 (21.9)

### Sociodemographic correlates of overweight and obesity

Table 2 presents the determinants of each obesity level. The odds of being obese were higher for those in lower socioeconomic group (SEIFA 5: Obese II OR = 2.46, 95% CI 1.85–3.28, Obese III OR = 3.79, 95% CI 2.53–5.68) compared to higher socioeconomic group. Respondents who are not in the labour force have 65% higher likelihood of being highest level of obese (95% CI 1.29–2.11). Middle age group (40–49 years old and 50–59 years old) showed higher likelihood of being overweight and obese compared to other age groups. On the other hand, higher education level and higher alcohol intake showed adverse relationship with obesity level. High education group had 53% (95% CI 0.36–0.61) and 58% (95% CI 0.29–0.59) lower likelihood of being obese II and obese III, respectively. The odds of being severe obese were significantly lower for those who were born in non-English speaking countries (Obese II OR = 0.29, 95% CI 0.21–0.41, Obese III: OR = 0.26, 95% CI = 0.17–0.41). Drinking alcohol more than 3 days per week had 70% lower likelihood of being obese III (95% CI = 0.22–0.41). In addition, we found that Indigenous status has a positive association with lower obesity level (Obese II OR = 1.68, 95% CI 1.09–2.60) but the association was not significant for Obese III (OR = 0.9, 95% CI 0.50–1.60).

**Table 2:** Determinants of obesity levels.

	Overweight	Obese I	Obese II	Obese III
	OR (95% CI)	OR (95% CI)	OR (95% CI)	OR (95% CI)
SEIFA 1 (highest)	Ref	Ref	Ref	Ref
SEIFA 2	1.04 (0.91–1.19)	1.19* (0.99–1.43)	1.13 (0.85–1.50)	1.67** (1.11–2.51)
SEIFA 3	1.29*** (1.12–1.49)	1.43*** (1.18–1.73)	1.52*** (1.14–2.01)	2.57*** (1.72–3.83)
SEIFA 4	1.19** (1.03–1.38)	1.47*** (1.21–1.78)	1.62*** (1.22–2.14)	2.93*** (1.98–4.34)
SEIFA 5 (lowest)	1.26*** (1.07–1.48)	1.80*** (1.47–2.20)	2.46*** (1.85–3.28)	3.79*** (2.53–5.68)
Male	Ref	Ref	Ref	Ref
Female	0.50*** (0.46–0.55)	0.65*** (0.57–0.73)	0.98 (0.82–1.16)	1.28** (1.02–1.60)
20–29	Ref	Ref	Ref	Ref
30–39	1.55*** (1.35–1.78)	1.53*** (1.28–1.84)	1.66*** (1.27–2.17)	1.88*** (1.35–2.61)
40–49	2.00*** (1.74–2.30)	2.32*** (1.94–2.79)	2.73*** (2.10–3.54)	3.59*** (2.62–4.93)
50–59	2.25*** (1.94–2.60)	2.74*** (2.28–3.28)	2.97*** (2.29–3.86)	2.89*** (2.08–4.01)
60–64	2.17*** (1.81–2.59)	2.40*** (1.92–3.00)	2.78*** (2.04–3.78)	1.79*** (1.18–2.70)
Low education	Ref	Ref	Ref	Ref
Middle education	1.04 (0.91–1.20)	0.92 (0.78–1.09)	0.88 (0.71–1.09)	0.95 (0.73–1.24)
High education	0.88 (0.76–1.04)	0.58*** (0.48–0.70)	0.47*** (0.36–0.61)	0.42*** (0.29–0.59)
Employed	Ref	Ref	Ref	Ref
Unemployed	0.93 (0.72–1.21)	1.09 (0.80–1.49)	1.14 (0.75–1.72)	1.10 (0.64–1.89)
Not in labour	0.79*** (0.69–0.90)	1.05 (0.90–1.23)	1.05 (0.85–1.30)	1.65*** (1.29–2.11)
Non-Indigenous	Ref	Ref	Ref	Ref
Indigenous	1.27 (0.93–1.75)	1.50** (1.06–2.13)	1.68** (1.09–2.60)	0.90 (0.50–1.60)
Australian	Ref	Ref	Ref	Ref
Main English-speaking country	0.86* (0.73–1.01)	0.82* (0.66–1.01)	0.97 (0.73–1.29)	0.85 (0.58–1.25)
Others	0.63*** (0.55–0.73)	0.41*** (0.33–0.50)	0.29*** (0.21–0.41)	0.26*** (0.17–0.41)
Married	Ref	Ref	Ref	Ref
Not married	0.83*** (0.74–0.92)	0.75*** (0.65–0.85)	0.85* (0.71–1.03)	1.06 (0.84–1.32)
New South Wales	Ref	Ref	Ref	Ref
Victoria	0.97 (0.86–1.10)	0.99 (0.85–1.17)	1.02 (0.81–1.28)	1.26 (0.94–1.70)
Queensland	0.99 (0.87–1.13)	0.99 (0.84–1.16)	0.97 (0.77–1.23)	1.10 (0.82–1.49)
South Australia	0.93 (0.77–1.11)	1.14 (0.92–1.42)	1.22 (0.90–1.65)	1.46** (1.02–2.10)
Western Australia	1.18* (0.99–1.40)	1.13 (0.89–1.42)	1.34* (0.98–1.83)	1.35 (0.89–2.06)
Tasmania	1.08 (0.82–1.42)	1.20 (0.87–1.66)	1.14 (0.73–1.77)	1.26 (0.72–2.17)
Northern Territory	1.07 (0.66–1.71)	0.89 (0.46–1.70)	1.16 (0.47–2.88)	1.29 (0.37–4.45)
Australian Capital Territory	1.05 (0.76–1.43)	1.56** (1.06–2.30)	1.51 (0.82–2.78)	1.72 (0.71–4.17)
Urban	Ref	Ref	Ref	Ref
Rural	1.07 (0.92–1.23)	1.06 (0.89–1.26)	1.20 (0.94–1.51)	0.96 (0.70–1.30)
No alcohol intake	Ref	Ref	Ref	Ref
Drink alcohol < 3 days per week	1.01 (0.90–1.13)	0.77*** (0.67–0.88)	0.76*** (0.63–0.92)	0.60*** (0.47–0.76)
Drink alcohol 3 + days per week	0.90* (0.79–1.01)	0.60*** (0.51–0.70)	0.42*** (0.34–0.54)	0.30*** (0.22–0.41)
Non smoker	Ref	Ref	Ref	Ref
Ex smoker	1.32*** (1.17–1.47)	1.65*** (1.44–1.90)	1.69*** (1.39–2.06)	1.49*** (1.16–1.91)
Current smoker	0.93 (0.82–1.06)	1.15* (0.98–1.35)	1.00 (0.79–1.25)	0.84 (0.63–1.12)

Regressions were adjusted for sex, age group, education level, household income, indigenous status, country of birth, marital status, Australian state, residential area, alcohol intake, and smoking.

***p < 0.01.

**p < 0.05.

*p < 0.1.

### Association between obesity, health service use, and work productivity

#### Health service use across percentiles of outcome variables

Table 3 describes the association between obesity levels and health service use. A higher level of obesity was associated with higher probability of using any GP (Obese III: OR = 1.42 95% CI 1.04–1.93), hospitalisation (Obese III: OR = 1.60 95% CI 1.22–2.09), as well as use of prescribed medication (Obese III: OR = 2.40, 95% CI 1.94–2.96).

**Table 3:** Distribution of health service use by obesity levels.

a. Distribution of GP visits by obesity levels							
	Any GP visit (n = 11,198)	Number of GP visit (n = 9434)					
		Mean	10th	25th	50th	75th	90th
Number of GP visit (normal weight)		5.32	1	2	3	6	12

	OR (95% CI)	Coef. (95% CI)	Coef. (95% CI)	Coef. (95% CI)	Coef. (95% CI)	Coef. (95% CI)	Coef. (95% CI)
Normal weight		Ref	Ref	Ref	Ref	Ref	Ref
Overweight	1.16** (1.02–1.31)	0.52*** (0.23, 0.80)	0.00 (− 0.09–0.09)	0.11** (0.00–0.22)	0.27*** (0.08–0.46)	0.46** (0.09–0.83)	0.94** (0.14–1.74)
Obese I	1.44*** (1.22–1.69)	1.44*** (1.08, 1.80)	0.00 (− 0.11–0.11)	0.46*** (0.32–0.59)	0.76*** (0.54–0.99)	1.63*** (1.17–2.09)	3.52*** (2.54–4.50)
Obese II	1.80*** (1.39–2.33)	1.77*** (1.26, 2.28)	0.00 (− 0.15–0.15)	0.39*** (0.20–0.58)	0.73*** (0.41–1.05)	1.43*** (0.80–2.07)	3.58*** (2.21–4.95)
Obese III	1.42** (1.04–1.93)	2.91*** (2.28, 3.54)	1.00*** (0.81–1.19)	0.70*** (0.47–0.94)	1.63*** (1.23–2.03)	3.23*** (2.43–4.03)	6.54*** (4.83–8.25)

b. Distribution of nights at hospital by obesity levels							
	Any hospitalisation (n = 11,199)	Number of nights at hospital (n = 1313)					
		Mean	10th	25th	50th	75th	90th
Number of nights at hospital (normal weight)		5.68	1	1	3	5	12

	OR (95% CI)	Coef. (95% CI)	Coef. (95% CI)	Coef. (95% CI)	Coef. (95% CI)	Coef. (95% CI)	Coef. (95% CI)
Normal weight	Ref	Ref	Ref	Ref	Ref	Ref	Ref
Overweight	1.25 (1.08–1.45)	0.03 (− 0.17, 0.22)	0.00 (0.00–0.00)	− 0.00 (− 0.24–0.24)	− 0.07 (− 0.61–0.47)	− 0.53 (− 1.84–0.78)	− 0.56 (− 6.56–5.44)
Obese I	1.45 (1.22–1.72)	0.11 (− 0.13, 0.36)	0.00 (0.00–0.00)	0.00 (− 0.28–0.28)	0.21 (− 0.41–0.83)	− 0.08 (− 1.59–1.42)	− 0.64 (− 7.53–6.25)
Obese II	1.33 (1.05–1.69)	0.21 (− 0.14, 0.55)	0.00 (0.00–0.00)	− 0.00 (− 0.37–0.37)	− 0.37 (− 1.21–0.47)	0.38 (− 1.67–2.42)	3.30 (− 6.06–12.65)
Obese III	1.6 (1.22–2.09)	1.24*** (0.81, 1.67)	0.00 (0.00–0.00)	0.00 (− 0.41–0.41)	0.51 (− 0.42–1.44)	1.97* (− 0.29–4.23)	7.30 (− 3.04–17.64)

c. Distribution of prescribed medication by obesity levels							
	Any prescribed medications (n = 11,203)	Number of prescribed medications (n = 4479)					
		Mean	10th	25th	50th	75th	90th
Number of prescribed medications (normal weight)		1.90	1	1	1	2	3

	OR (95% CI)	Coef. (95% CI)	Coef. (95% CI)	Coef. (95% CI)	Coef. (95% CI)	Coef. (95% CI)	Coef. (95% CI)
Normal weight	Ref	Ref	Ref	Ref	Ref	Ref	Ref
Overweight	1.27*** (1.15–1.41)	0.17*** (0.10, 0.25)	0.00 (0.00–0.00)	0.00 (− 0.11–0.11)	− 0.00 (− 0.14–0.14)	0.23** (0.01–0.45)	0.27 (− 0.12–0.67)
Obese I	1.84*** (1.63–2.08)	0.54*** (0.44, 0.63)	0.00 (0.00–0.00)	0.00 (− 0.12–0.12)	− 0.00 (− 0.16–0.16)	0.59*** (0.34–0.84)	1.30*** (0.84–1.76)
Obese II	2.02*** (1.70–2.39)	0.70*** (0.57, 0.84)	0.00 (0.00–0.00)	0.00 (− 0.16–0.16)	− 0.00 (− 0.21–0.21)	0.76*** (0.43–1.09)	1.34*** (0.74–1.94)
Obese III	2.40*** (1.94–2.96)	1.07*** (0.91, 1.24)	0.00 (0.00–0.00)	0.00 (− 0.19–0.19)	1.00*** (0.75–1.25)	1.23*** (0.84–1.62)	2.20*** (1.49–2.91)

Regressions were adjusted for sex, age group, education level, household income, indigenous status, country of birth, marital status, Australian state, residential area, alcohol intake, and smoking.

***p < 0.01.

**p < 0.05.

*p < 0.1.

Amongst those with normal weight and had any health service use, the estimated mean number of GP visit, nights at hospital, and prescribed medications were higher than their median (GP visit: 5.32 vs. 3, nights in hospital: 5.68 vs. 3, prescribed medication: 1.90 vs. 1), illustrating the positively skewed nature of the distributions.

Table 3a shows the relationships between obesity level and GP visits by health service use quantile. An increase in BMI (from normal weight to overweight, obese I, obese II, and obese III) was associated with a higher likelihood of GP visits and a higher number of GP visits in every quantile group. The effect of obesity on GP visits was found to be greater in the upper tail of health service use distributions than in the lower tail. For example, the coefficient for obese II was 0 (95% CI − 0.15–0.15) for the 10th percentile, and 3.58 (95% CI 2.21–4.95) for the 90th percentile). The coefficient for obese III was 1.0 (95% CI 0.81–1.19) for the 10th percentile and 6.54 (95% CI 4.83, 8.25) for the 90th percentile.

Table 3b presents the relationship between obesity level and number of nights at a hospital. Number of nights in hospital was positively associated with obesity II and III at the 75th and 90th percentiles of the distribution while it was negatively associated with a lower level of obesity. However, these associations were not statistically significant.

The association between obesity level and number of prescribed medications is presented in Table 3c. Higher obesity level was associated with higher likelihood of an increasing number of prescribed medications compared to normal weight at the upper tail of the distribution. The effect of obesity was greater at the 75th and 90th percentiles of the distribution compared to the lower tail. For example, the coefficient for obese II was 0.76 (95% CI 0.43–1.09) for the 75th percentile and 1.34 (95% CI 0.74–1.94) for the 90th percentile. The coefficient for obese III was 1.23 (95% CI 0.84–1.62) for the 75th percentile and 2.20 (95% CI 1.49–2.91) for the 90th percentile.

#### Work productivity by percentiles

As shown in Table 4, a higher level of obesity was associated with higher probability of taking any sick leave compared to normal weight (Obese II: OR = 1.45, 95% CI 1.19–1.76, Obese III: OR = 1.62 95% CI 1.25–2.09). Of respondents who took any sick leave, the impact of obesity levels on number of sick leave was larger at the higher quantiles compared to lower quantiles. The coefficients for obese II was 0.52 (95% CI − 0.11–1.14) for the 10th percentile and 1.91 (95% CI 0.96–2.86) for the 75th percentile. The coefficient for obese III was 0.61 (95% CI − 0.19–1.41) for the 10th percentile and 2.83 (95% CI 1.62–4.03) for the 75th percentile. The association between high level of obesity and number of sick leave was not statistically significant at the 90th percentile of the distribution.

**Table 4:** Distribution of sick leave days by obesity levels.

	Any sick leave (n = 9375)	Number of sick leave days (n = 4905)					
		Mean	10th	25th	50th	75th	90th
Number of sick leave days (normal weight)		6	1	2	4	6	10

	OR (95% CI)	Coef. (95% CI)	Coef. (95% CI)	Coef. (95% CI)	Coef. (95% CI)	Coef. (95% CI)	Coef. (95% CI)
Normal weight	Ref	Ref	Ref	Ref	Ref	Ref	Ref
Overweight	1.22*** (1.10–1.35)	0.01 (− 0.54, 0.55)	0.04 (− 0.30–0.39)	− 0.00 (− 0.19–0.19)	0.18 (− 0.09–0.44)	0.35 (− 0.17–0.87)	0.17 (− 0.80–1.13)
Obese I	1.32*** (1.15–1.51)	0.86** (0.16, 1.55)	0.38* (− 0.07–0.82)	− 0.00 (− 0.24–0.24)	0.66*** (0.32–1.00)	1.27*** (0.61–1.94)	1.67*** (0.43–2.90)
Obese II	1.45*** (1.19–1.76)	0.89* (− 0.10, 1.88)	0.52 (− 0.11–1.14)	1.00*** (0.66–1.34)	0.91*** (0.43–1.40)	1.91*** (0.96–2.86)	0.93 (− 0.82–2.69)
Obese III	1.62*** (1.25–2.09)	0.85 (− 0.41, 2.11)	0.61 (− 0.19–1.41)	1.00*** (0.57–1.43)	1.39*** (0.78–2.01)	2.83*** (1.62–4.03)	1.53 (− 0.70–3.76)

Regressions were adjusted for sex, age group, education level, household income, indigenous status, country of birth, marital status, Australian state, residential area, alcohol intake, and smoking.

***p < 0.01.

**p < 0.05.

*p < 0.1.

## Discussion

### Principal findings

Overall, the prevalence of overweight and obesity was 35.0% and 27.6%, respectively, among Australian residents aged 15 years and above during the period of 2017–2018. After adjusting for socio-demographic factors, low socioeconomic status was associated with overweight and high level of obesity while high education group was associated with a lower likelihood of being obese. Generally, a higher level of obesity was associated with higher probability of health service use, and work productivity loss. Regarding quantile analyses, using quintile regression methods, this study showed that the high level of obesity had significantly increased number of GP visits and prescribed medications across the outcome distributions, In terms of work productivity loss, the impact of obesity levels on number of sick leave was larger at upper tiles of distributions. These findings suggested that the impacts of overweight and obesity on health service use could be higher for obese individuals mainly because these individuals may have more healthcare needs.

### Comparison with existing literature

We found that the prevalence of overweight and obesity was 35.0% and 27.6%, which is consistent with population data from Australia^1,24^. Our findings that lower income and educational attainment were associated with higher probability and levels of obesity is also in line with literature in Australia and other high-income countries^25,26^.

Higher levels of obesity were associated with increased number of GP visits and number of prescribed medications, which has been found in studies from other high-income countries^9,11,12,27,28^. The quantile regression analysis show that such association was more profound among those at upper tiles of distribution (i.e. among those use more GP service and prescribe more medications). This is in line with a study used quantile regression to examine association between BMI categories with health costs^29^. The finding of increased health service use is indicative of the direct costs of overweight and obesity in Australia, with annual estimates of between $2 Billion and $3.8 Billion^24,30,31^.

Overweight and obesity were associated with increased likelihood and number of sick leave days taken, and the effect was larger at upper tiles of distributions. Though focused on a sample from defence force in Australia, the author also found that productivity losses from full days off work were higher in the obese subgroup compared with the normal cohort^17^. Our finding is also in line with a border literature on the indirect costs of obesity on productivity loss^32–34^, with obesity believed to incur productivity costs of almost $5 Billion annually in Australia^31,35,36^.

### Strengths and limitations

This is the first study to explore healthcare use and productivity loss associated with obesity using nationally representative data in Australia. Besides, this is a novel study to estimate the effect of obesity applying quantile regression which reflects the variations in the impact of obesity across the outcome distributions.

However, there are some limitations. First, we use self-reported data for BMI which may be biased due to measurement error. An existing study indicates that obese people are likely to underestimate weight^37^. In addition, self-reporting of health service use and productivity is likely to cause recall error as the respondents are required to remember and report information from the last 12 months. Second, the outcomes of the impact of obesity on health service use and productivity in this study might be underestimated, as the survey data was not collected from patients with severe illness or those admitted to hospital. Third, healthcare utilisation and productivity loss were not specifically due to illness or disability associated with obesity, and could have included other unrelated reasons and conditions such as acute illnesses and physical injury. Fourth, we used alcohol consumption based on weekly frequency but not on intensity, thus, it may not reflect the actual amount of alcohol consumption. Further, our analysis on work productivity only looked at number of sick leave days as an indicator due to the availability of question. Thus, the results might not capture the full picture of the burden of obesity. Other indicators of work productivity such as wage loss or presenteeism should be taken into account for future studies. Another limitation of our study is that our estimation may be biased as a result of omitting confounding factors that may be associated with outcomes, such as individuals' local environment and genetic factors, which are not captured in the HILDA survey. Lastly, the interpretation of causal effect might be limited because of the use of cross-sectional study design. Future studies need to consider using longitudinal data to observe a long-term effect of obesity on outcomes.

### Policy implications

These findings highlight the significant variance in the demands that differing severities of overweight and obesity can place on health care systems, as well as its implication for employment productivity on both a personal and population level. This raises the question as to the appropriateness of direct and indirect cost estimates for obesity based on linear regression models. While this may capture mean estimated costs, they do not reflect the implications at the different quantiles of the distribution of outcomes.

Our results of the quantile regression analyses demonstrate the associations between obesity or overweight largely varied at different quantiles. The impact of obesity is larger for those with a higher degree of obesity and with greater use of health service and lower work productivity. A likely account for this result was that obese individuals suffer double economic burdens of increasing use of health services and reduced work productivity.

It is evident from our findings that policy approaches to address obesity and its impacts must be two-fold; firstly, addressing the prevalence of overweight and obesity in Australia, and secondly in reducing its association with adverse health service and productivity outcomes. A range of policy approaches are needed in order to confer both benefits, targeting obesity on both an individual and a population or workforce level. There are a number of broad measures to reduce the prevalence of excess weight which are supported by the literature; taxes on low nutrition foods and sugar sweetened beverages, while politically difficult have been modelled to reduce obesity and improve productivity^35,38,39^, as have investments in urban design to facilitate greater physical activity^40,41^. On an individual level, there is evidence for utilisation of bariatric surgery to both reduce excess weight and its impact on employees wellbeing, as well as reducing barriers to employment^42,43^.

It is also necessary that interventions and programs target those who already suffer from overweight and obesity, in order to improve their wellbeing and occupational performance. There is encouraging evidence that workplace-based health interventions can see improvements in health and productivity before a noticeable decline in overweight and obesity^44–46^. Evidence supports targeted interventions by Primary care practitioners^35,47^, however a lack of implementation of established guidelines for obesity management in Australia has hampered the effectiveness of this^48,49^. Failure to focus on both the management and prevention of obesity will see poor returns in the form of ongoing costs for both individuals and wider societal costs (Supplementary Table 1).

## Supplementary Information

Supplementary Information.

## Data Availability

Data is publicly available from Household, Income and Labour Dynamics in Australia Survey website: https://melbourneinstitute.unimelb.edu.au/hilda.

## Abbreviations

BMI: Body mass index
HILDA: Household, income, and labour dynamics Australia
HRQoL: Health-related quality of life
SEIFA: Socio-economic indexes for areas
OLS: Ordinary least squares
