# Author Correction: The association between obesity, health service use, and work productivity in Australia: a cross-sectional quantile regression analysis

**DOI:** 10.1038/s41598-023-35911-0

**Published:** 2023-05-31

**Authors:** Marie Ishida, Monique D’Souza, Yang Zhao, Tianxin Pan, Will Carman, Tilahun Haregu, John Tayu Lee

**Affiliations:** 1 School of Population and Global Health, Nossal Institute for Global Health, University of Melbourne, Melbourne, Australia; 2 The George Institute for Global Health at Peking University Health Science Center, Beijing, China; 3 The George Institute for Global Health at University of New South Wales, Sydney, Australia; 4 Health Economics Unit, Centre for Health Policy, Melbourne School of Population and Global Health, University of Melbourne, Melbourne, Australia; 5 Department of Primary Care and Public Health, Faculty of Medicine, Imperial College London, London, UK; 6 Department of Health Service Research, Faculty of Medicine, Australian National University, Canberra, Australia

Correction to: *Scientific Reports* 10.1038/s41598-023-33389-4, published online 24 April 2023

In the original version of this Article Tianxin Pan was incorrectly affiliated with ‘School of Population and Global Health, Nossal Institute for Global Health, University of Melbourne, Melbourne, Australia’.

The correct affiliation is listed below.

Health Economics Unit, Centre for Health Policy, Melbourne School of Population and Global Health, University of Melbourne, Melbourne, Australia

The original Article has been corrected.

