# Supplementary material for: The association between obesity, health service use, and work productivity in Australia: a cross-sectional quantile regression analysis
Source: Sci Rep. 2023 Apr 24;13:6696. doi: 10.1038/s41598-023-33389-4 (PMC10126067; doi:10.1038/s41598-023-33389-4)
Supplement: Supplementary file 1 — Supplementary Information. [file 41598_2023_33389_MOESM1_ESM.docx]

Appendix

**Appendix Table 1. Definition of outcome variables**

| Outcome variables | HILDA survey question |
| --- | --- |
| **GP visits**  Any GP visits (0: no, 1: yes)  Number of GP visits (numeric) | *Approximately how many times have you seen a family doctor or another GP about your health in the last 12 months?* |
| **Nights at hospital**  Any overnight stay at hospital  (0: no, 1: yes)  Number of nights at hospital (numeric) | *Altogether, in the last 12 months, how many nights did you stay in hospital?* |
| **Prescribed medication**  Any prescribed medication  (0: no, 1: yes)  Number of prescribed medication (numeric) | *Altogether, how many different prescription medications do you take on a regular basis?* |
| **Number of sick leave days**  Any sick leave taken  (0: no, 1: yes)  Number of sick leave (numeric) | *How many weeks or days did you spend on paid sick leave?* |

**Appendix Figure 1. Sample flow chart**

**HILDA wave 17**

N=17570

Respondents

- Aged 20-65years

- BMI≥18.5

N=11373

**Final sample**

N=11211

**Health service use**

GP visit N=11198

Nights at hospital N=11199

Medication N=11203

**Work productivity**

Sick leave N=9375

**Removed**

Those who are not employed

N=1836

**Removed**

Those with missing values on covariates

N=162

**Removed**

N=6197

**Appendix Figure 2. Association between number of GP visit and obesity levels**


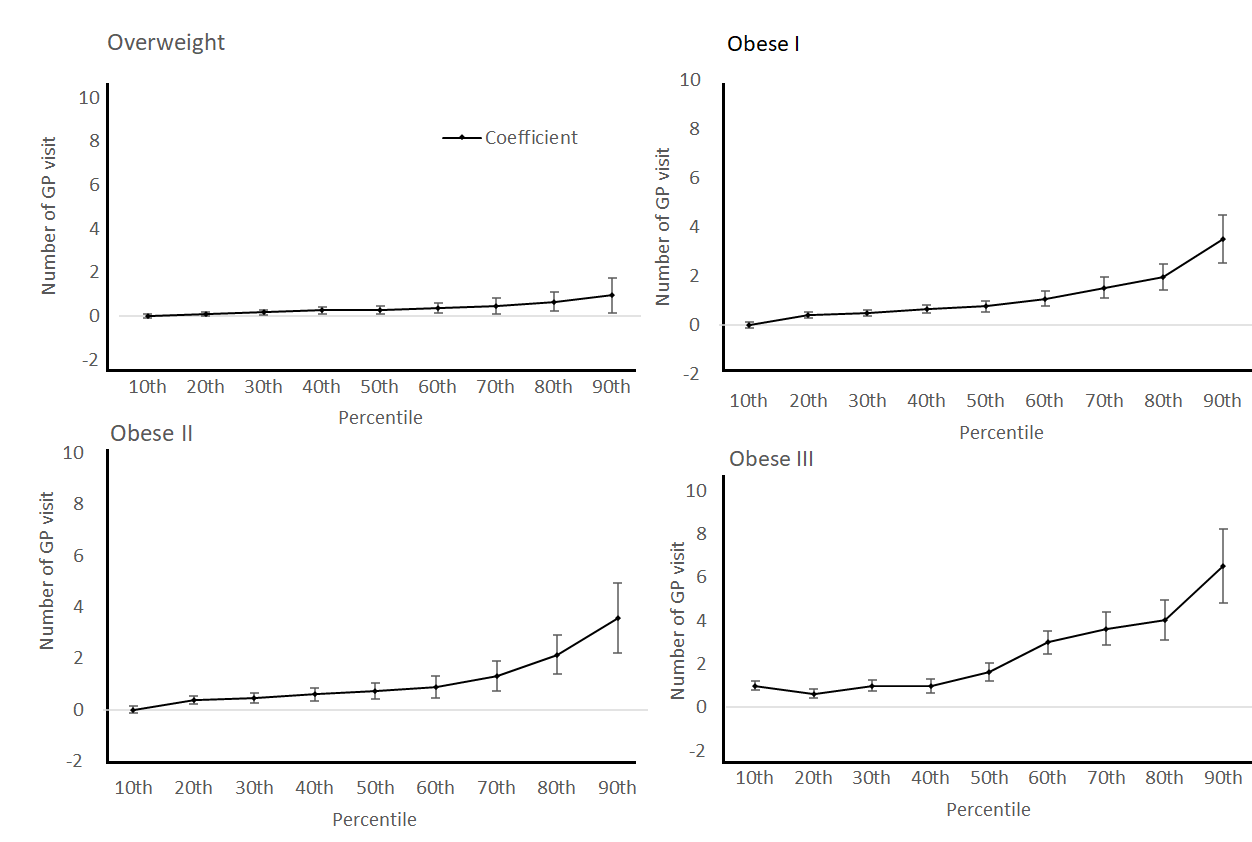


*Note*: GP=General Practice

**Appendix Figure 3. Association between number of nights at hospital and obesity levels**


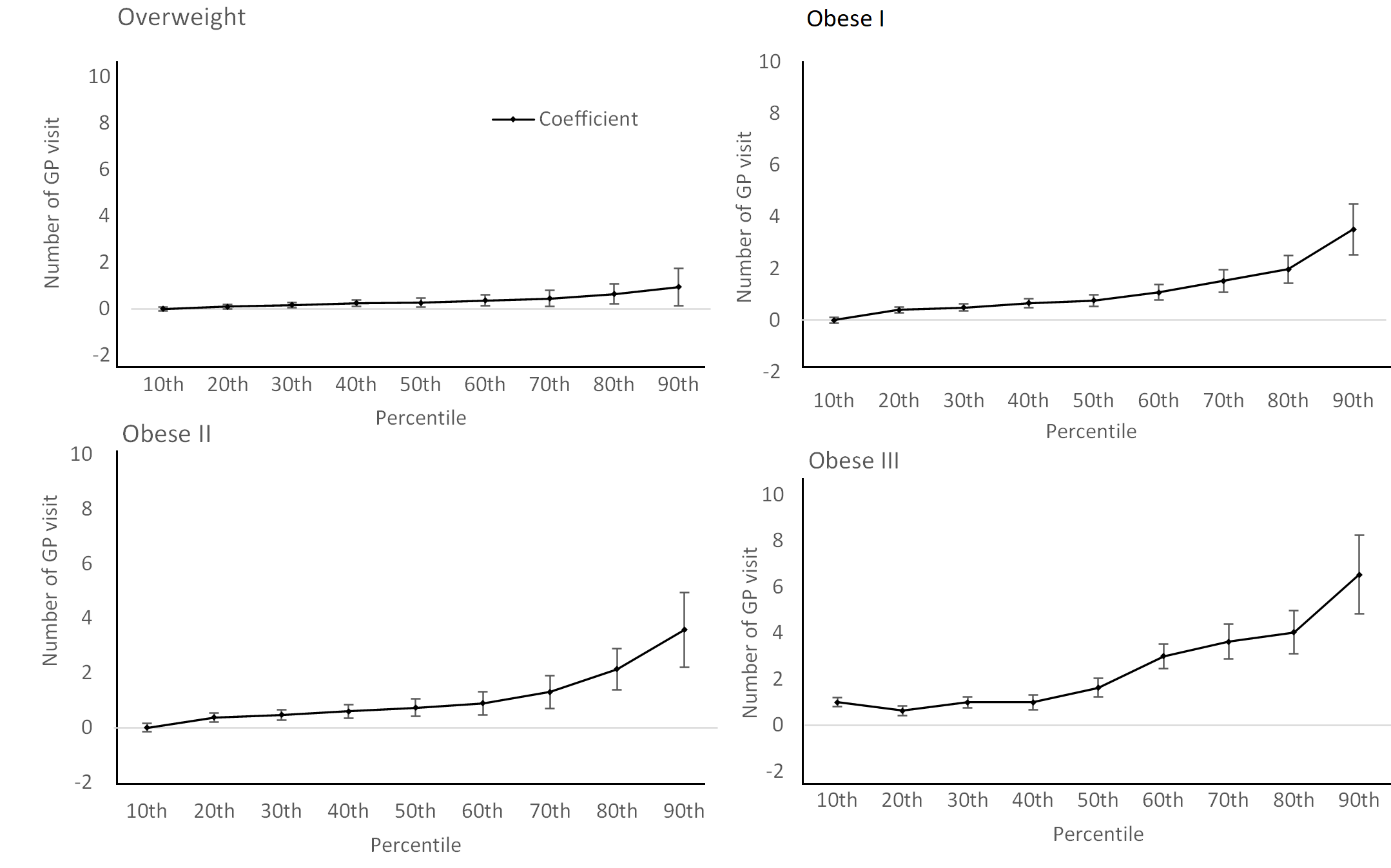


**Appendix Figure 4. Association between number of medication and obesity levels**

**Appendix Figure 5. Association between number of sick leave days and obesity levels**
